# Supplementary material for: Multiple Autonomous Cell Death Suppression Strategies Ensure Cytomegalovirus Fitness
Source: Viruses. 2021 Aug 27;13(9):1707. doi: 10.3390/v13091707 (PMC8473406; doi:10.3390/v13091707)
Supplement: Supplementary file 1 [file viruses-13-01707-s001.zip › viruses-1311688-supplementary.pdf]

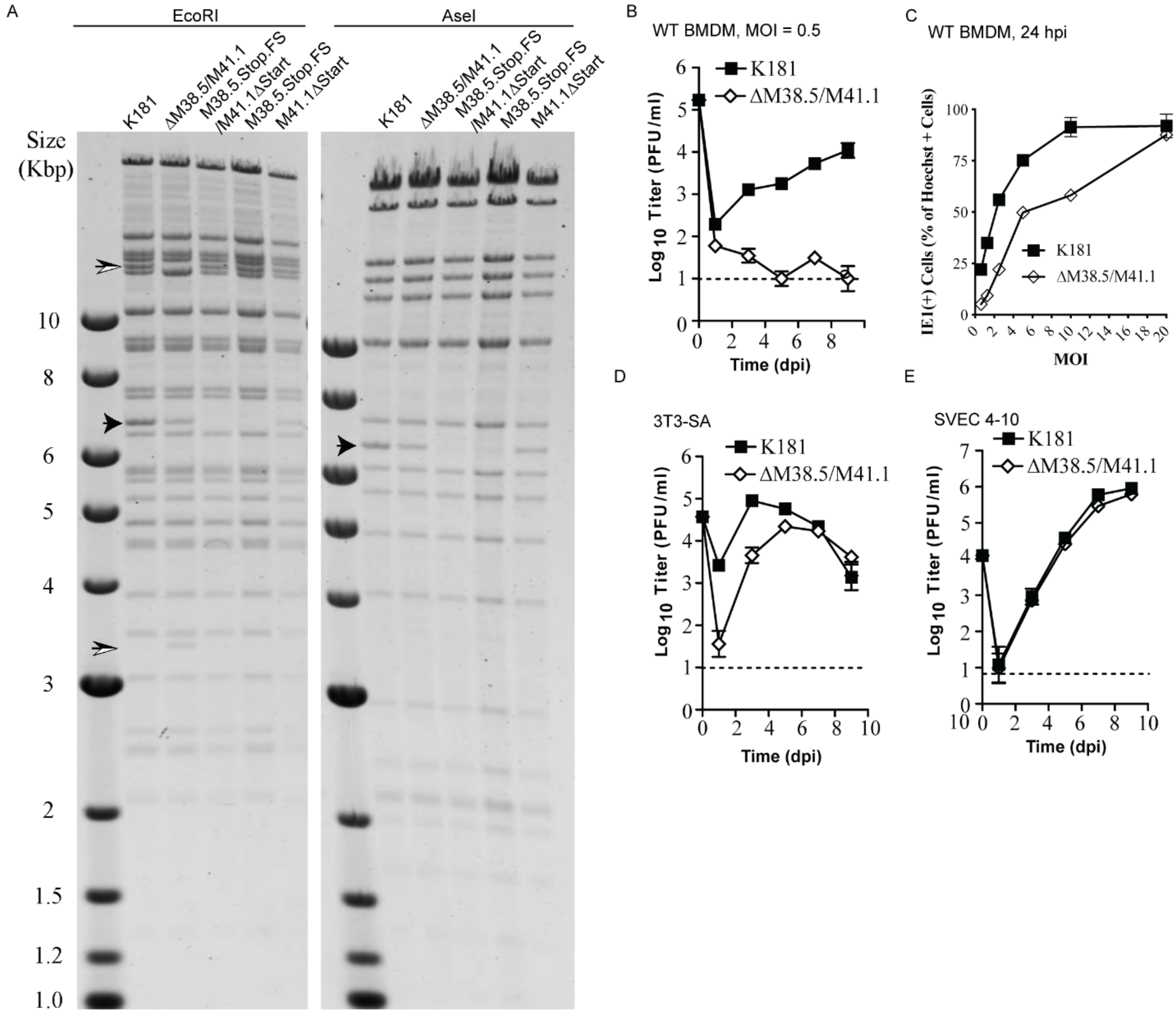

Figure S1. Mutagenesis of M38.5 and M41.1. (**A**) The genomic integrity of the K181 and mutant viruses analyzed by electrophoretic separation of EcoRI- and AseI-digested BAC DNA on 0.6% agarose gels. Arrowheads indicate predicted changes in the EcoRI RFLP pattern of ΔM38.5/M41.1-BAC. A 13 Kbp band generated by EcoRI digestion was disrupted upon insertion of the 2.9 Kbp Kan/SacB cassette (total size of 15.9 Kbp). The Kan/SacB cassette created an additional EcoRI restriction site, dividing the 15.9 Kbp fragment into 3.3 Kbp and 12.6 Kbp bands. The loss of the 13 Kbp band and appearance of the 3.3 Kbp band are marked by the black-and-white arrow heads. The black only arrowheads indicate the presence of the plasmid pSIM6 encoding the λ red recombineering functions. pSIM6 digestion by EcoRI and AseI produced a linear 6.7 Kbp band in the appropriate lanes (lanes 1, 2, and 5). (**B**,**C**) Replication kinetics (**B**) and infectivity (**C**) of K181 or ΔM38.5/M41.1 virus in WT BMDM at indicated MOIs and times. MCMV-encoded IE1 expression was used as marker for infectivity. (**D**,**E**) replication kinetics of K181 or ΔM38.5/M41.1 in 3T3-SA (**D**) and SVEC4-10 (**E**) cells over the course of time indicated. BMDM were infected at MOI = 0.5, SVEC4-10 and 3T3-SA cells were infected at MOI = 0.3. Infected cells with media were collected at 1, 3, 5, 7 and 9 dpi. For each panel, data shown are pooled results from at least three experiments where error bars indicate standard error and mean.

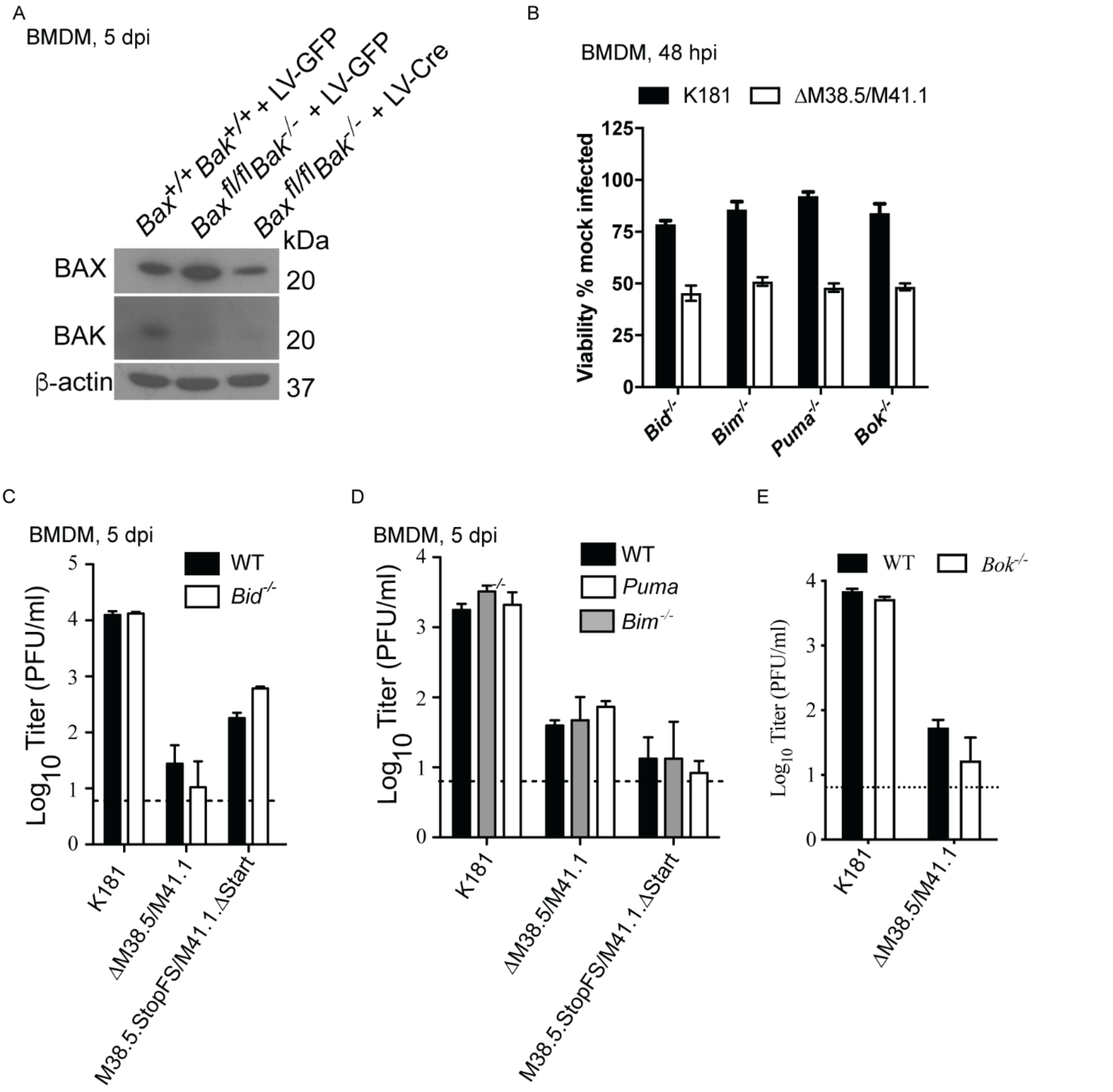

Figure S2. M38.5 and M41.1 target BAX and BAK to facilitate MCMV replication. (**A**) Immunoblot analysis of cells with transduction settings described in (**A**) to detect BAK (~20 kDa) and BAK (~20 kDa) expression with loading control β-actin (38.5 kDa). Uninfected cells were harvested for immunoblot at the same time infected cells were collected for titer assessment. (**B**) Viability of WT, *Bid*$^{-/-}$, *Bim*$^{-/-}$, *Puma*$^{-/-}$ and *Bok*$^{-/-}$ cells infected with K181 or ΔM38.5/M41.1 (MOI = 10) assessed at 48 hpi. (**C**–**E**) Replication of K181, ΔM38.5/M41.1 or M38.5.StopFS/M41.1ΔStart viruses in WT and *Bid*$^{-/-}$ (**C**), WT, *Puma*$^{-/-}$, and *Bim*$^{-/-}$ BMDM (**D**), as well as WT and *Bok*$^{-/-}$ BMDM (**E**) at 5 dpi (MOI = 0.05). For each experiment, data shown are pooled results from at least three experiments. No significant changes in ΔM38.5/M41.1 infection were observed for any of the mutant cells when compared by Wilcoxon matched pairs signed rank test. Error bars indicate standard error and mean for each dataset.

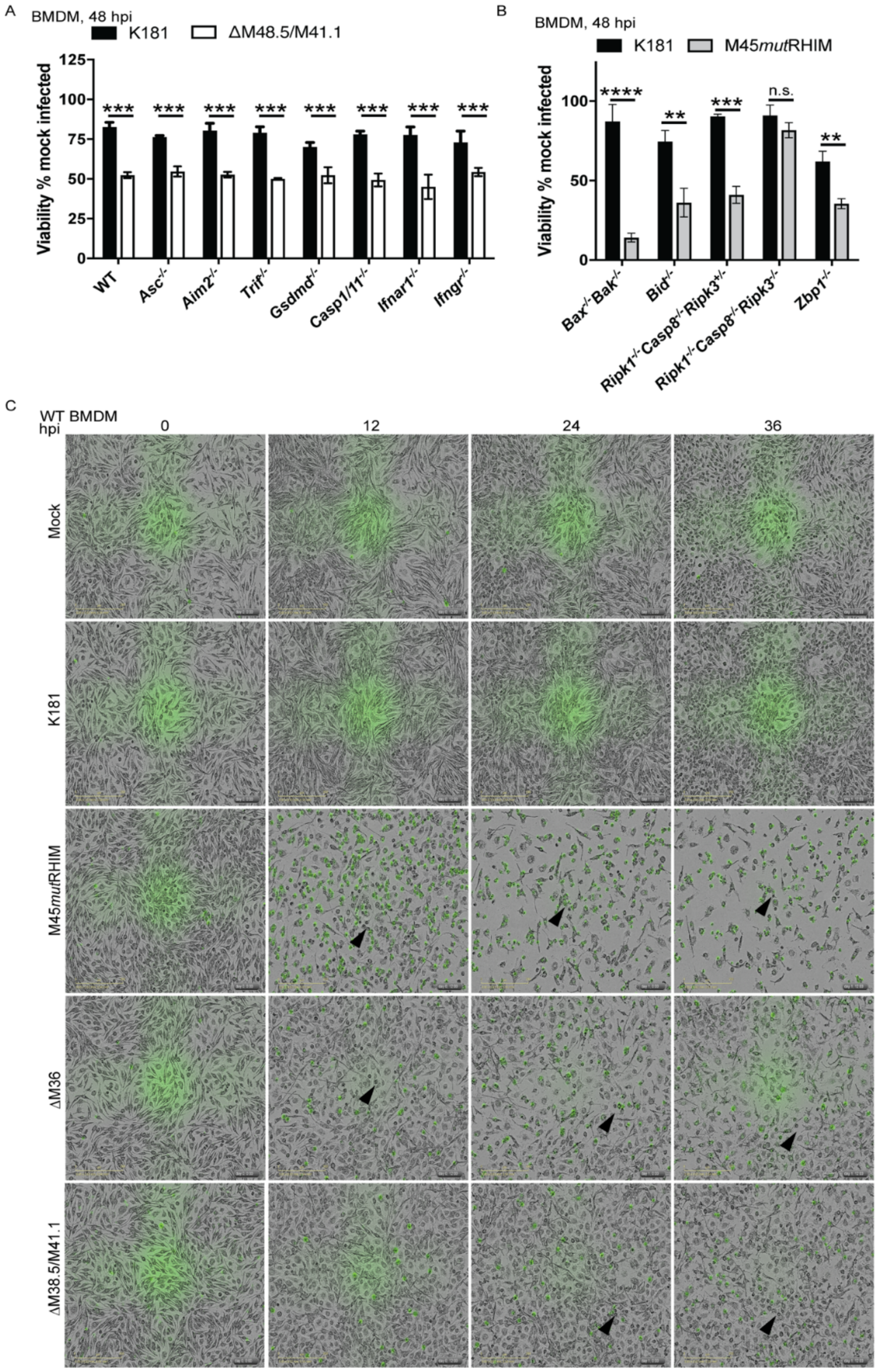

Figure S3. Requirements for mutant MCMV-induced apoptosis and necroptosis. (**A**) Viability of BMDM from mice of indicated genotypes infected with K181 or ΔM38.5/M41.1 (MOI = 10) at 48 hpi. Each bar represents the pooled data from at least three experiments. (**B**) Viability of BMDM of indicated genotypes infected with K181 or M45*mut*RHIM (MOI = 10) at 48 hpi. Each column represents the pooled data from at least three experiments. Horizontal bars indicate the two groups being compared in each setting utilizing Wilcoxon matched pairs signed rank test. Error bars indicate standard error and mean for each dataset. ** is $p < 0.01$; *** is $p < 0.001$; **** is $p < 0.0001$; n.s. is non-significant. (**C**) Representative light microscopy images (lower left scale bar = 200 µM) of WT BMDM infected with indicated viruses at indicated times. Time stamp from microscope is included in lower right corner of each image. Cell death is measured by inclusion of cell-impermeable dye (Sytox Green) as cellular membrane loses integrity due to execution of death signaling. Diffused circular green signal (detected in middle of lanes of fields without signal from cells) is a nonspecific consequence of the microscope light filter and camera.

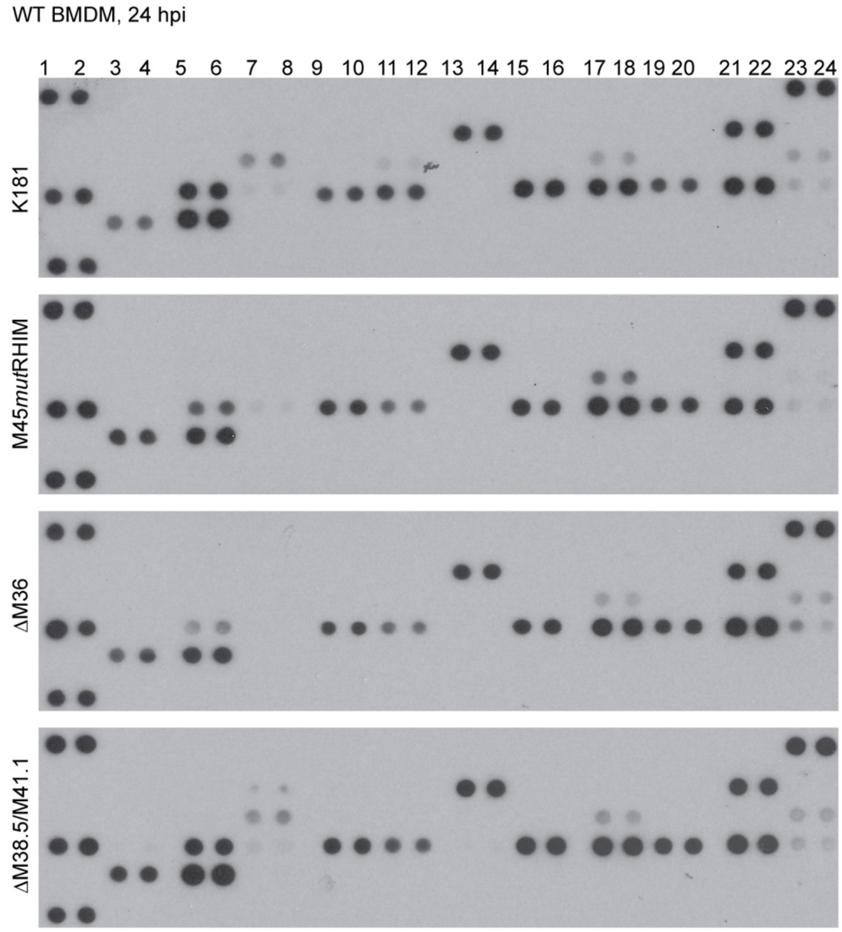

A	Ref-1											Ref-2
B	BLC	C5	G-CSF	GM-CSF	I-309	CCL11	siCAM-1	IFNG	IL-1α	IL-1β	IL-1ra	IL-2
C	IL-3	IL-4	IL-5	IL-6	IL-7	IL-10	IL-13	IL-12 p70	IL-16	IL-17	IL-23	IL-27
D	IP-10	I-TAC	KC	M-CSF	JE	MCP-5	MIG	MIP-1α	MIP-1β	MIP2	RANTES	SDF-1
E	TARC	TIMP-1	TNF-α	TREM-1								
F	Ref-3											Neg

Figure S4. Dot blot proteome profiler array of supernatant from infected cells (related to Figure 3F). Cell-free supernatants from WT BMDM, infected collected 24 hpi with indicated viruses, were assessed by protein profiler array. (A–F) represent 6 rows where every two dots are replicates of distinct proteins. The names of the proteins are reflected in the map provide at lowest panel.

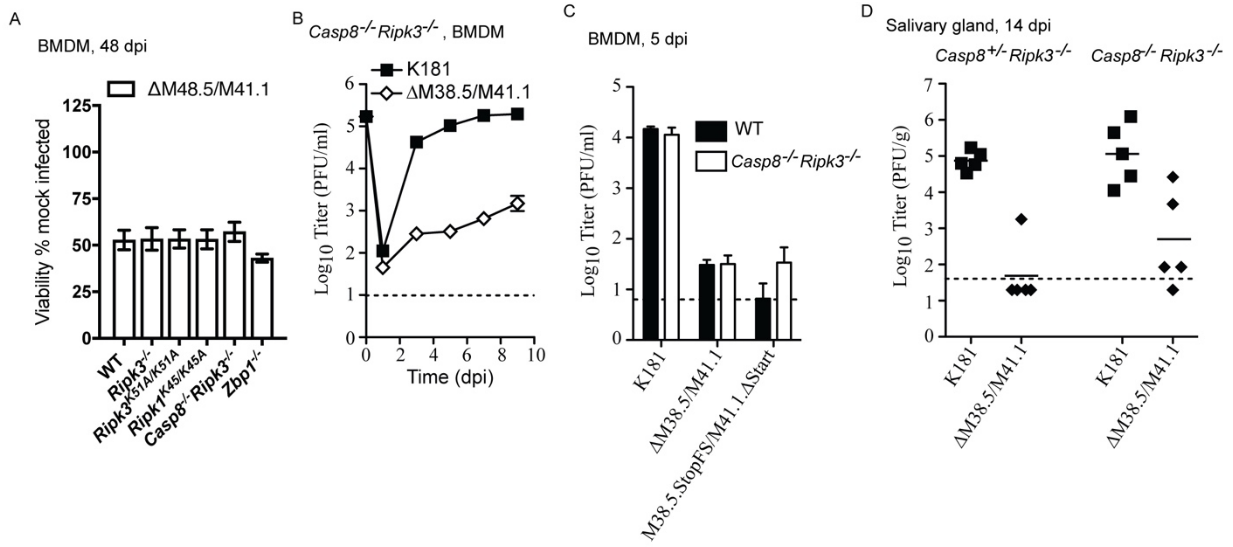

Figure S5. Ripoptosome components are dispensable for ΔM38.5/M41.1 MCMV replication and cell death. (**A**) Viability of WT or *Ripk3*$^{-/-}$, *Ripk3*$^{K51A/K51A}$, *Ripk1*$^{K45A/K45A}$, *Casp8*$^{-/-}$*Ripk3*$^{-/-}$ and *Zbp1*$^{-/-}$ BMDM infected with K181 or ΔM38.5/M41.1 (MOI = 10) at 48 hpi. Each bar represents pooled data from at least three experiments. (**B**) Replication kinetics of K181 or ΔM38.5/M41.1 virus in *Casp8*$^{-/-}$*Ripk3*$^{-/-}$ BMDM infected at MOI = 0.5 over 9 days. Replication was measured as described in Figure S1. (**C**) Replication of K181 or indicated mutant MCMV in WT or *Casp8*$^{-/-}$*Ripk3*$^{-/-}$ BMDM infected at MOI = 0.5 for 5 d. For each experiment (**A**–**C**), data shown are pooled results from at least three experiments where error bars indicate standard error and mean for each dataset. (**D**) Replication of K181 and ΔM38.5/M41.1 in salivary glands of littermate *Casp8*$^{+/-}$*Ripk3*$^{-/-}$ or *Casp8*$^{-/-}$*Ripk3*$^{-/-}$ mice at 14 dpi. Mean is indicated for each dataset.
